# A case of ruptured infective coronary artery aneurysm

**DOI:** 10.1186/s40792-017-0347-6

**Published:** 2017-06-07

**Authors:** Kayo Sugiyama, Katsuhiko Matsuyama, Keita Maruno, Satoshi Takahashi, Masahiko Kuinose, Rena Nagashima, Hitoshi Ogino

**Affiliations:** 1 Department of Cardiovascular Surgery, Tokyo Medical University Hospital, 6-7-1 Nishishinjuku, Shinjuku-ku, Tokyo, 160-0023 Japan; 2 Department of Cardiac Surgery, Aichi Medical University Hospital, Aichi, Japan; 3 Department of Cardiovascular Surgery, Kawasaki Medical School Hospital, Okayama, Japan; 4 Department of Radiology, Tokai University Tokyo Hospital, Tokyo, Japan

**Keywords:** Coronary artery aneurysm, Coronary artery bypass grafting (CABG), Inflammatory aneurysm

## Abstract

Infective coronary artery aneurysm is extremely rare and ruptured aneurysm is life-threatening. We report a case of ruptured coronary artery aneurysm, which was successfully treated by the patch closure technique and coronary artery bypass grafting. Pathological examination revealed purulent inflammation in the aneurysmal wall. Prompt diagnosis and appropriate treatment were essential.

## Background

Coronary artery aneurysm is a rare disease. Recently, it has been detected more frequently because of advancements in imaging technology. Its main complication is thromboembolic events; however, the rate of rupture remains unknown [1].

We encountered a case of ruptured coronary artery aneurysm with purulent inflammation, which was successfully treated by patch closure of the aneurysm and coronary artery bypass grafting (CABG). Although the natural history of infective coronary artery aneurysm remains unknown, early recognition and prompt surgical treatment are necessary to prevent fatal complications.

## Case presentation

**Fig. 1:**
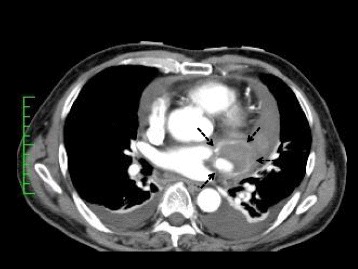
Computed tomography showing pericardial effusion (*solid arrow*) and a coronary artery aneurysm (*dotted arrow*) with an enhanced wall

The patient was a 67-year-old man whose chief complaints were shortness of breath and high fever. He had been treated for hypertension at a local hospital and never had any obvious past histories associated with Kawasaki disease or other infectious diseases. His coronary artery aneurysm had already been detected by chest computed tomography (CT). The aneurysm diameter was more than 2 cm. Just before admission, the patient suffered from worsening of dyspnea and sudden chest pain. An emergency CT scan showed moderate pericardial effusion and enhancement of the aneurysmal surface (Fig. 1).

**Fig. 2:**
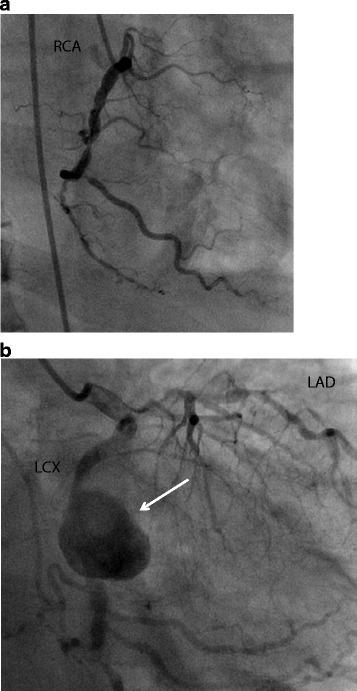
**a** Right coronary artery angiography showing severe stenosis. **b** Left Coronary artery angiography showing a saccular aneurysm of the left circumflex artery (*solid arrow*) and severe stenosis

On admission, the patient’s clinical data were as follows: blood pressure, 129/76 mmHg; pulse rate, 83/min; body temperature, 38 °C; oxygen saturation, 94% under 2 L/min oxygen supply. The laboratory data showed the following: severe inflammatory changes; C-reactive protein level, 19 mg/dL; white blood cell count, 10 × 10^3^/μl. Chest X-ray revealed mild cardiomegaly. Electrocardiography indicated no remarkable ischemic changes. Echocardiography showed moderate pericardial effusion and reduced left ventricular function which left ventricular ejection fraction was 50%. Coronary angiography revealed severe triple vessel disease (Fig. 2a, b) and a saccular aneurysm originating from the circumflex coronary artery (Fig. 2b), total occlusion of the left anterior descending artery and right coronary artery, and severe stenosis of the left circumflex artery.

**Fig. 3:**
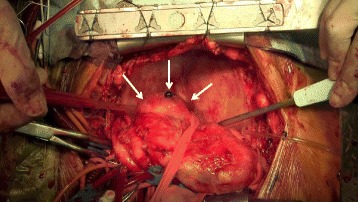
A giant coronary artery aneurysm (*solid arrow*) originating from the circumflex coronary artery

During the surgery, we found a small amount of bloody pericardial effusion and a large hematoma. Neither the walls of the aneurysm nor the pericardium appeared to be acutely inflamed. The bleeding from the ruptured aneurysm had already stopped (Fig. 3).

**Fig. 4:**
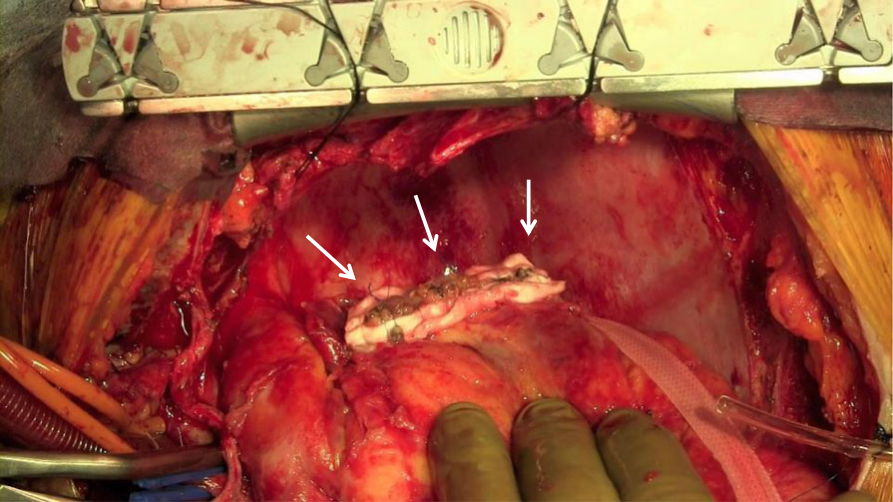
Ligated aneurysm using mattress sutures and felt sheets (*solid arrow*)

After achieving cardiac arrest by antegrade cold blood cardioplegia, the aneurysm was opened. The ostia of the proximal and distal parts of the aneurysm were closed using mattress sutures with a felt sheet, and the aneurysmal wall was ligated with a felt patch using 4–0 monofilament mattress sutures (Fig. 4). After ligation of the aneurysmal wall, CABG to the left anterior descending artery using the left internal mammary artery, and to the posterolateral branch and the obtuse marginal branch using a saphenous vein graft in a sequential anastomosis fashion was performed. The revascularization to right coronary artery was not performed because it was a small artery and the perfusion area was limited.

The postoperative course was uneventful, and the patient was discharged on postoperative day 14. The antibiotic therapy, i.e., cefazolin sodium, was administrated preoperatively and had been given for a week postoperatively to avoid surgical site infection and recurrence of aneurysmal infection. The patient showed clinical improvement with no signs and symptoms of infection, and the C-reactive protein decreased 19 to 0.5 mg/dL in 2 weeks. Therefore, the additional antibiotic therapy was not continued. Postoperative CT scan showed no abnormal flow into the aneurysm, and all the bypass grafts were patent.

**Fig. 5:**
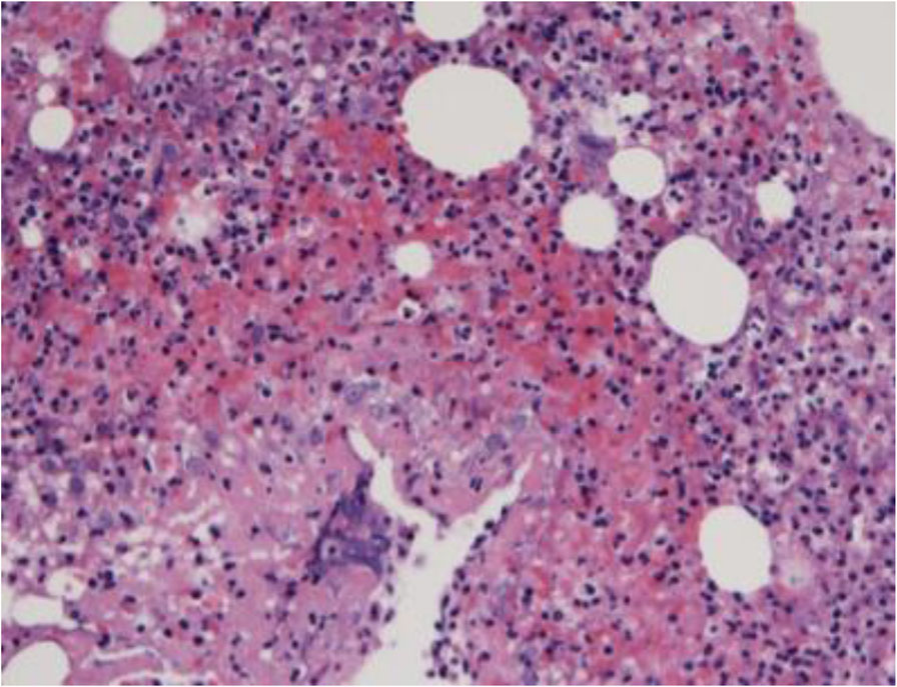
Pathological examination of the aneurysmal wall showing severe inflammatory changes (invasion of neutrophils and lymph cells, granulation tissue, necrosis, and abscess)

Blood culture was negative, and pathological examination revealed severe inflammatory changes, namely, invasion of neutrophils and lymph cells, granulation tissue, necrosis, and abscess in the aneurysmal wall; however, there were no signs of bacterial colony (Fig. 5). It seemed that lymphocyte filtration was rather much; however, the pathologist concluded severe inflammation due to infection because of neutrophils and abscess in the aneurysmal wall. The culture of the aneurysmal wall was negative, and the blood examination results had improved dramatically after the surgical repair. The patient remains well and free from any cardiac events in 3 years.

### Discussion

The pathology of coronary artery aneurysm was first described by Morgagni in 1761 [2], and the first clinical case was reported by Bourgon in 1812 [2]. In recent years, the diagnosis rate of coronary artery aneurysm has been increasing because of advancements in imaging technology, with an incidence of 1–5% in patients who undergo coronary artery angiography [1, 3, 4].

Coronary artery aneurysm is defined as an artery whose diameter is 1.5 times larger than the normal diameter [1]. If the diameter is larger than 20 mm, this condition is defined as a giant coronary artery aneurysm [5]. Aneurysms more often occur in the right coronary artery and less frequently in the left main coronary artery [1]. The main complication of aneurysms is thromboembolism (e.g., myocardial infarction) [1].

Some of the causes of coronary artery aneurysm include atherosclerosis, Kawasaki disease, iatrogenic complications, vasculitis, syphilis, and other infectious diseases [1]. The etiology, however, varies geographically. In Europe and North America, the causes are atherosclerosis, congenital heart disease, and Kawasaki disease in 50, 17, and 10% of patients, respectively [4]. In contrast, in Japan and China, the main cause of aneurysms is Kawasaki disease [2, 6]. However, the present case never had any past history of Kawasaki disease, and other infectious diseases were not detected.

On the other hand, mycotic coronary artery aneurysms are extremely rare, occurring in less than 0.5% of all infective endocarditis cases [7, 8]. Several mechanisms may be involved in their pathogenesis as follows: embolic occlusion and sterile infarction of the vasa vasorum, direct bacterial invasion of the arterial wall, and injury from deposition of immune complexes in the arterial wall. The present patient showed no signs of infective endocarditis on admission and during the operation. However, he suffered from a high fever and his laboratory data indicated severe inflammatory changes on admission. The pathological examination revealed purulent inflammation. Based on these considerations, infection possibly occurred in the coronary artery wall leading to the development of a large aneurysm.

A few studies have reported different probabilities of aneurysmal rupture. Daoud et al. reported that 12% of 57 coronary artery aneurysms ruptured [9]; however, their report was an autopsy review. In the Coronary Artery Surgery Study Registry, Swaye et al. did not detect any cases of ruptured aneurysm in 978 patients [3]. The natural history of an infective coronary artery aneurysm is more unclear than that of a normal aneurysm; however, there is a higher tendency of rupture in a mycotic aneurysm [8]. Prompt diagnosis and treatment should be performed for an infective coronary artery aneurysm. Otherwise, Swaye et al. reported that most patients with a coronary artery aneurysm had significant stenosis or occlusion. According to these reports, coronary artery angiography should be performed to detect a fatal coronary artery stenosis or occlusion in every patient with coronary artery aneurysm. Because the present case developed moderate pericardial effusion and severe coronary artery disease, in addition, stopping of the bleeding was not detected on arrival; the patient underwent emergency surgical treatment.

There are various surgical strategies to repair aneurysms such as interposition grafting, simple aneurysmal resection, and isolation of the aneurysm with concomitant CABG [10]. If there are active, acute, and uncontrolled infectious signs, interposition grafting close to the lesion would be dangerous [11]. Thus, ligation, excision, and distal coronary bypass would be appropriate in the present case. It remains controversial whether these procedures should be performed with or without cardiopulmonary bypass and with or without cardiac arrest. Li et al. normally used cardiopulmonary bypass with moderate hypothermia for aneurysm resection [2] to avoid cardiac ischemic events. Otherwise, there are some reports in which the aneurysms were treated by off-pump CABG [12]. The advantage of this method is its minimal invasiveness. In the present case, we performed CABG using cardiopulmonary bypass to stabilize the hemodynamics, and we closed the aneurysm under cardiac arrest to prevent distal embolization.

Although covered stent implantation is another option for a ruptured aneurysm [13], we did not choose this procedure because the patient showed severe infective signs and required several revascularizations for severe coronary artery diseases. Moreover, we cannot use a covered stent for a ruptured coronary artery aneurysm in Japan.

## Conclusions

We successfully performed a surgical treatment for a ruptured coronary artery aneurysm. The natural history of coronary artery aneurysm, particularly inflammatory aneurysm, remains unknown. However, prompt diagnosis and appropriate treatment are necessary. Coronary artery angiography should be performed in every patient with coronary artery aneurysm to detect coronary artery disease.

## Abbreviations

CT: Computed tomography
CABG: Coronary artery bypass grafting
